# Potential Use of Salivary Markers for Longitudinal Monitoring of Inflammatory Immune Responses to Vaccination

**DOI:** 10.1155/2016/6958293

**Published:** 2016-02-28

**Authors:** Pei Wen Lim, Johan Garssen, Elena Sandalova

**Affiliations:** ^1^Immunology Platform, Nutricia Research, Singapore 138671; ^2^Division of Pharmacology, Utrecht Institute for Pharmaceutical Sciences, Faculty of Science, Utrecht University, 3584 CS Utrecht, Netherlands; ^3^Immunology Platform, Nutricia Research, 3584 CT Utrecht, Netherlands

## Abstract

Vaccination, designed to trigger a protective immune response against infection, is a trigger for mild inflammatory responses. Vaccination studies can address the question of inflammation initiation, levels, and resolution as well as its regulation for respective studied pathogens. Such studies largely based on analyzing the blood components including specific antibodies and cytokines were usually constrained by number of participants and volume of collected blood sample. Hence, blood-based studies may not be able to cover the full dynamic range of inflammation responses induced by vaccination. In this review, the potential of using saliva in addition to blood for studying the kinetics of inflammatory response studies was assessed. Saliva sampling is noninvasive and has a great potential to be used for studies aimed at analysing the magnitude, time course, and variance in immune responses, including inflammation after vaccination. Based on a literature survey of inflammatory biomarkers that can be determined in saliva and an analysis of how these biomarkers could help to understand the mechanisms and dynamics of immune reactivity and inflammation, we propose that the saliva-based approach might have potential to add substantial value to clinical studies, particularly in vulnerable populations such as infants, toddlers, and ill individuals.

## 1. Introduction

Inflammation is part of the body's natural immune defenses reactivity. Inflammation is essential for a proper immune response to protect the body against infectious insults; however, excessive inflammation can cause significant damage. Thus, an appropriate control of inflammation is essential for maintaining health. Moreover, in most cases we do not know how much inflammation is too much and when should it stop. Inflammation occurs when the body is injured or infected. Such conditions trigger an immune response, which leads to raised levels of inflammatory markers (within minutes or hours) that in turn activate and recruit immune cells [1]. At a later stage, the immune cells will participate in the resolution of inflammation and in the healing process.

**Figure 1:**
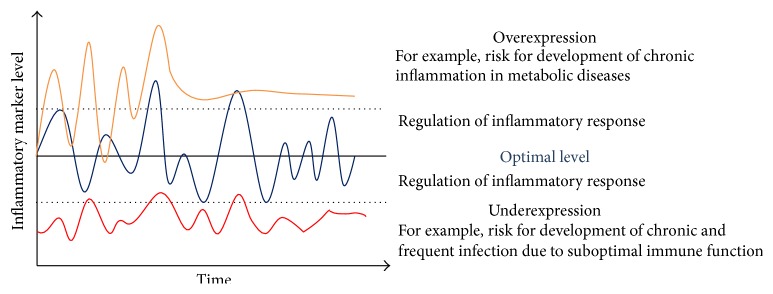
Illustration of general paradigm of the regulation of inflammatory response.

The general paradigm of the regulation of inflammatory responses is illustrated in Figure 1. Inflammatory markers fluctuate within the boundaries of a normal healthy range (dotted lines in Figure 1) to support a healthy balanced immune response. However, a disruption in the regulation of inflammatory processes such as a too slow or delayed return to the basal state; or a constantly high level of inflammatory response after an insult might lead to the progression of a chronic inflammatory state. There is growing evidence that an elevation in systemic concentrations of proinflammatory cytokines, such as interleukin-1 (IL-1), IL-6, and tumor necrosis factor-alpha (TNF-*α*), or an elevation in systemic concentrations of acute-phase reactant C-reactive protein (CRP) is associated with risk of developing metabolic-syndrome related diseases, including type 2 diabetes, and even cardiovascular diseases, neurodegenerative diseases, cancer, asthma, and obesity in healthy individuals [2–5]. On the other hand, underexpression or an insufficient inflammatory response can lead to a higher susceptibility for infections [6]. Hence, studying inflammation is an important topic of current medical and immunological research programs [7, 8]. However, since most approaches use blood to study inflammatory responses they are limited since blood drawing is an invasive procedure which discourage participants from joining the study and which should always be under a thorough ethical review.

Studying inflammation triggered by natural pathogens is challenging and does not come without risks. Natural infection is unpredictable due to variation in the degree of antigenic exposure and virulence factors as well as timing of exposure. As an alternative, the development of an inflammatory response can be studied in healthy individuals by using vaccination, which provides an experimental setting mimicking an infection. Vaccines aim to induce memory B and T cells which would be able to respond fast to a real infection. B cells produce pathogen-specific antibodies and T cells destroy the pathogen directly. A drawback of such approaches is the induction of inflammation that is necessary to create a stable memory response, however, which makes the vaccinated individual feel unwell. How much inflammation is necessary for a strong immune response is not clear. In the ideal scenario, vaccination should induce just enough inflammation to trigger the immune response but not causing the individual to feel ill. In this review, we do not focus on the specific antibody or T cell response, but instead we aim to discuss the noninvasive approaches for studying the inflammatory response induced by vaccination.

## 2. Methods 

Three strategies were used to obtain relevant literature to support our proposal to use a noninvasive approach to measure inflammatory response:

1. Studying inflammation induced by vaccination: Literature search was performed in PubMed and all articles which are presented in full text have been used. All articles were manually screened to identify studies addressing the magnitude, time course, and variance of inflammatory response after vaccination. Key search terms included “vaccination,” “immunization,” and “inflammatory markers”. Twelve articles (age range from preterm to elderly) were selected for review (Table 1).
2. Literature search was performed in PubMed to obtain overview of studies that have used saliva for measuring inflammatory markers. The titles and the abstracts were manually screened and twenty-nine relevant articles were selected.
3. The Google and the Biocompare.com database were used to provide an overview for commercially available kits for measuring biomarkers in saliva (Table 2).

Whereas this is not a systematic review, our goal is to provide a broad overview and to highlight the potential of using noninvasive methods for immunomodulation studies.

## 3. Vaccination as an Immune Challenge 

Vaccination is an ideal model of an artificial infection to induce the immune response without eliciting clinical symptoms of disease. It is a way of triggering the immune response by administrating a controlled and safe dosage of an antigen that is either an inactivated or weakened infectious agent. This agent activates the immune system and induces the protection against specific infectious diseases. Moreover, vaccination induces a mild systemic inflammatory response involving a small increase in acute-phase proteins and proinflammatory cytokines. The localized inflammatory response is initiated at the injection site upon vaccination and rapidly triggered by tissue macrophages to secrete cytokines (IL-1, IL-6, and TNF-*α*). The vast numbers of inflammatory mediators produced, for example, IL-6, flow out of the inflammatory site into the circulation and are subsequently accompanied by a slight systemic inflammatory response. The systemic response increases the level of leukocytes and upregulates the synthesis of hormones such as adrenocorticotropic hormone (ACTH) that stimulates secretion of glucocorticoids (such as cortisol). IL-6, IL-1, and cortisol have a central role in inducing the hepatic synthesis of the acute-phase proteins, such as CRP, in the liver. Such inflammatory responses after vaccination are mostly detectable in plasma or serum. Furthermore, these inflammatory cytokines and mediators derive from the local vasculature that originates from the carotid arteries can outflow into saliva and could be detected in saliva sample as well [9].

Vaccination in young children is typically the first planned inflammatory challenge [10]. Now it is also acknowledged as being able to train innate immunity in order to exert a stronger immune response following a subsequent challenge [11, 12]. Most vaccination protocols require repeated administration of the vaccine and it would be valuable to understand how the inflammatory-immune response changes from the first dose of vaccine to another to evaluate the safety and effectiveness of vaccine response. Research showed that the immunization of newborns and infants with live bacillus Calmette-Guerin (BCG) and measles vaccines induce Th1 responses, proinflammatory cytokines which activate or recruit immune cells to the injection site. This enhances the memory characteristics of NK cells and provides a beneficial effect in reducing mortality of neonates due to nonspecific immune stimulation that provides protection against other infections [11, 13, 14]. On the other hand, the inactivated Diphtheria-Tetanus-Pertussis (DTP) and Hepatitis B (HepB) vaccines may have nonspecific deleterious effects, as they have been reported associated with sepsis in young children in high-mortality countries [14–17]. Sepsis is associated with a cytokine storm which is caused by a nonspecific overactivation of the immune system. Thus, it is essential to examine factors other than specific antibody titres after vaccine administration. However, as to which level of inflammation is necessary and enough to induce a strong and long-lasting immune response to the vaccine is currently not known.

IL-6 and CRP are the most common markers aimed at determining the inflammatory response to the HepB vaccine in infants in order to prevent sepsis or other severe adverse reactions after injection. A study observed CRP elevation after 24 hours of HepB vaccine injection in 70 healthy term infants [18]. In another study, a peak response of IL-6 and CRP was seen after 12 hours and 36 hours after a set of immunizations with DTwP, HIB (Hemophilus influenza type B), HBV, and IPV (inactive poliovirus) vaccines in preterm infants [19]. Thus, vaccination-triggered inflammation in children might be particularly interesting to study in more detail, including closer monitoring of the time of the inflammation peak and the time the inflammation declines. In our view, closer monitoring of inflammation could be achieved by incorporating measurement of proinflammatory cytokines and CRP in a noninvasive approach such as analysis of saliva. As saliva collection is more acceptable by the majority and accessible compared to other sampling methods (stool and urine). This close monitoring could provide an answer to the questions of what level of inflammation is necessary for achieving immune protection.

## 4. Inflammatory Response to Vaccination

Many studies have used vaccine challenges to study responses of the inflammatory immune system. Doing so enables studying the range of dynamic variations of inflammation markers in plasma, which is important to define whether an individual is a high or low inflammation responder [20–23]. The inflammatory response elicited by vaccination is substantially milder and more transient than seen in infectious illness, making it a desirable and safer setting for studies in all population groups. Most importantly, there is much better control clinically of the antigen administered in the study; for example, a known amount of specific antigen is used. Moreover, the magnitude and time patterns of the inflammatory response from vaccination are predictable, which makes it easier to define the time points for sample collection [19].

A number of studies that have examined inflammatory responses to vaccination are summarized in Table 1. All these studies have used blood for the measurements. The main inflammatory markers that indicate the level and the kinetics of inflammation after vaccination in these studies are CRP, IL-1, IL-6, and TNF-*α* [21]. IL-1*β* is one of the most prominent proinflammatory cytokines that promotes the release of IL-6 and TNF-*α* [24]. In addition, IL-6 and CRP are closely related: IL-6 is secreted by immune cells (T cells and macrophages) upon infection or injury and induces CRP production in the liver; and CRP is a well-known inflammatory marker commonly used for monitoring sepsis, cardiovascular conditions, postsurgical complication, and systemic inflammation response [25]. According to the American Heart Association (AHA) guidelines, levels of CRP in blood above 5 or 10 mg/L are used to identify an acute-phase activity or acute inflammation. Such a high level of CRP for the short-term, as an innate immune response to pathogenic challenge, is necessary to protect us from infectious disease [26, 27]. In contrast, having CRP levels stable over time above 3 mg/L is considered to be chronic low-grade inflammation which contributes to the risk for development of metabolic diseases like cardiovascular diseases, Type 2 diabetes (T2D), and obesity [27, 28]. Thus, it is important to capture CRP responses to vaccination and correlate it to protective antibody titers.

It is worth noting that these inflammatory markers are evoked by different stimuli (i.e., different vaccines mentioned in the studies) and measured in blood across different time courses. Therefore, the reported peak level for each marker may not be comparable from one study to another. As shown in Table 1, vaccination against* Salmonella typhi* was frequently used in the studies to induce transient systemic inflammation, followed by influenza vaccination. The* S. typhi* and influenza vaccinations are known to be relatively safe for use in adults to induce a low-grade proinflammatory stimulus. Both vaccines are approved by the United States Food and Drug Agency to protect against food poisoning and flu, respectively [20]. Through studies of these vaccines, we have seen that IL-6 appears in blood prior to CRP. It should be noted that IL-6 is the inducer of CRP production; thus this time course is expected. The level of IL-6 gradually increased within 3–24 hours after injection, whereas CRP usually appeared at least 12 hours after injection. However, IL-6 could still be detected several weeks after vaccination in elderly, which is indicative that inflammation control in elderly is impaired [29]. In contrast to IL-6, the peak level in CRP has been demonstrated in the longer time course (by days) studies ranging between 24 and 48 hours [23, 30]. The production of IL-1 and TNF-*α* in response to vaccination did not exhibit consistency in these studies (Table 1).

It is worth noting that the number of participants in these studies ranged from 8 to 89, which is a small sample size to reach significant conclusions. Perhaps the invasive approach of drawing blood may have hindered the study recruitment and limited the choice of time points of sampling. A tighter and longer monitoring of inflammatory response to vaccination is needed with a higher number of participants to answer the questions of inflammation initiation, resolution, and control. Adopting a noninvasive sampling method such as saliva collection would help to overcome the small sample size issue and could increase the number of time points of sampling.

## 5. Limitations of Current Approaches

Many inflammation studies have been performed in different study populations with different vaccines. Serum or plasma samples have been evaluated at different time intervals after vaccination, either hourly or daily. The variation in sample timing may preclude capturing the peak of the inflammation responses and is unlikely to cover the full recovery to baseline levels. Additionally, inflammation level is also affected by other confounding factors such as, gender, age, and nutritional and metabolic profile of individuals that subsequently alter the vaccine immune response [31–33]. These factors could alter the pattern of inflammatory response to a vaccination. As mentioned above the kinetics of an IL-6 response were different in the elderly compared to adult study. Some studies selected only male individuals and did not compare to females. Thus, to obtain a full understanding of the kinetics of inflammatory events, it is necessary to have more effective sampling time-points such as to conduct a tighter and longer time-course study with a higher number of participants so that all applicable confounding factors could be taken into account. However, this approach would be challenging to perform as such research would require frequent blood draws in multiple time-points (hourly and extended to days) in vulnerable populations, such as infants, elderly, or diseased individuals [34, 35]. Furthermore, numerous times of needle pricking can be stressful for study participants. Moreover, many studies have shown that stress (measured by cortisol) could directly impact the inflammatory parameters, such as IL-6 [36–38]. Thus, these results would have to be interpreted taking into account the level of stress that is induced by the procedure itself. Hence, noninvasive approaches such as collections of urine, stool, and saliva could help overcome the fear and discomfort of blood draws during sampling in clinical research and could thus be recommended for kinetics in inflammatory response studies. Stool and urine collections can still present a challenge for study participants who are reluctant to perform the collection due to embarrassment, hygiene-related concerns, or worry about invasion of privacy [39]. Unlike stool and urine collections, saliva collection is by far most acceptable by study participants as it is quicker and requires minimal handling procedure.

The focus of the present review is to provide information on which inflammatory biomarkers can be determined in saliva and how these salivary biomarkers could contribute to the study of vaccination efficacy and safety. For a general review regarding salivary inflammatory markers in response to acute stress, see [17].

## 6. Use of Saliva in Clinical Research

Saliva is secreted by salivary glands which are composed of 99% water and 1% of electrolytes, proteins, mucus, hormones, and antibacterial components such as secretary immunoglobulin A (sIgA) and lysozyme [40]. The functions of saliva are more extensive than moistening food, producing bolus, and lubricating soft tissue in the esophagus; they also initiate digestion of carbohydrates and clear bacteria for oral health protection.

Saliva is a hypotonic fluid; its water and electrolytes components are derived largely from the local capillary bed via intracellular diffusion according to the osmotic gradient into salivary lumen whereas proteins and small components from the blood enter saliva by passive diffusion from the capillaries surrounding the salivary glands. The excessive constituents of blood in the systemic circulation such as drugs, hormones, and proteins will outflow to saliva via transcellular route (passive diffusion and active transport) and via a paracellular route passing through capillary wall, interstitial space, basal cell membrane, and cytoplasm of the salivary glands, as well as luminal cell membrane [41, 42]. Thus, saliva comprises pooled constituents from blood and the constituents that are locally produced in the mouth. With this unique context, saliva can serve as an effective indicator of both local and systemic biological activity [43–45]. However, the level of most serum constituents present in saliva is about 300–3000 times lower than that in the plasma [40]. Thus, there is a need to determine which serum constituents are measurable in saliva and their correlation between serum and saliva.

Owing to technological advances over the past decade, use of saliva as the measurement tool has become a popular alternative to blood (the gold standard) [46]. Saliva is nowadays used mainly for diagnosis of periodontal and other oral diseases [47]. Multiple research efforts are being dedicated to expand the use of saliva for the studies of systemic diseases and for monitoring of hormones and other drug levels in the body [48]. Analysis of studies' data suggests that saliva could provide important clinical screening and/or diagnostic information for diseases, such as asthma, cancer, and diabetes [49–51].

The rise of saliva usage in clinical research is due to the advantage that it can be collected noninvasively, which is safer and more feasible than drawing blood in infants [52], toddlers, adolescents, and elderly patients [53]. In addition, the procedures of saliva collection and processing are modest and cost effective for screening large sample size compared to blood and are thus also useful in field studies [9, 54].

## 7. Detection of Inflammatory Mediators in Saliva 

Several components in saliva such as glucose, insulin, cortisol, adipokines, CRP, and inflammatory cytokines have been used in many risk-stratification studies as an indicator of inflammation, stress, type 2 diabetes, and cardiovascular diseases in at-risk populations in all age ranges [55–57]. Saliva samples are mostly collected by the unstimulated passive drool method or the oral swab method [58]. Several factors such as salivary flow rate, circadian rhythm, type of salivary gland and salivary stimulus, diet, physiological status, age, and gender as well as method of collection can affect the sensitivity and the results of detection [45, 58–60]. For instance, many of the salivary cytokines are inversely proportional to salivary flow rate whereas CRP and cortisol are unaffected by the salivary flow rate. Salivary cortisol is in equilibrium with free cortisol in the plasma [61]. Free cortisol in the blood passes through membranes mainly by passive diffusion to appear in all bodily fluids, including blood, urine, sweat, semen, and saliva. Thus, salivary cortisol level is flow rate independent and it is directly reflected by the serum cortisol level. Additionally, IL-6 and cortisol levels fluctuate by circadian rhythm. They are therefore sensitive to sampling conditions such as time during the day (morning versus evening) when the sample is collected. In general, cortisol levels rise in the morning after awakening and fall during the day with a steep decline in cortisol levels exhibited across the day. A recent study has shown the diurnal pattern of salivary IL-6 and its relationship with psychosocial stress in healthy young adults [62]. The results showed that high salivary IL-6 levels detected in the morning are inversely associated with a lower cortisol awakening response. This finding supports the evidence that stress shapes the regulatory mechanisms of the hypothalamic-pituitary-adrenal axis (HPA) and should be taken into account when studying inflammatory responses.

## 8. Correlation between Saliva and Plasma

Salivary biomarkers can be measured with commercially available immunoassay kits (Table 2). Most employ enzyme-linked immunosorbent assays (ELISAs) technique for a single biomarker. For measuring multiple biomarkers, it is recommended to use the multiplex suspension array technology, which creates possibilities of measuring several cytokines and proteins simultaneously within the same small volume sample [63, 64]. Salimetrics LLC (Carlsbad, CA), a leading salivary research company, showed that 7 out of 19 salivary markers correlated with serum in their tests (Table 2).

A research study showed that 27 cytokines detected in blood, including IL-1*β*, IL-1 receptor agonist, IL-2, IL-4, IL-5, IL-6, IL-7, IL-8, IL-9, IL-10, IL-12, IL13, IL-17, eotaxin, basic fibroblast growth hormone (FGH), growth-colony stimulating factor (G-CSF), granulocyte-macrophage colony-stimulating factor (GM-CSF), interferon-gamma (IFN-*γ*), interferon-inducible protein 10 (IP-10), monocyte chemotactic protein-1 (MCP-1), macrophage inflammatory proteins- (MIPs-) 1alpha, MIP-1beta, platelet-derived growth factors- (PDGF-) BB, tumor necrosis factor- (TNF-) alpha, and vascular endothelial growth factor (VEGF) can be detected in healthy adult saliva as well [45]. However, in this study only 3 out of 27 cytokines (IL-6, IFN-*γ*, and MIP-1*β*) from saliva samples collected by passive drool method showed significant correlation (*p* < 0.05) with the levels measured in blood samples. It should be noted that the times of collection for such correlation study are essential at present as how long it takes for an induced blood marker to appear in saliva is not known. In addition, salivary flow rate and total salivary protein should be established in salivary analysis studies [65]. Total salivary protein can be used as marker for plasma protein leakage as a consequence of inflammation process [66].

Another correlation study of 16 inflammatory biomarkers in saliva and blood was performed in healthy and depressed adolescents [63]. The study found a higher detection rate in saliva compared to serum, in which 14 out of 16 biomarkers were detected in more than 80% of saliva samples except for IL-10 and INF-*α*2. Interestingly, this study did not find any correlation in the level of IL-6 in saliva and serum, which is incongruent with the previous study mentioned above [45]. However, it is difficult to compare study to study as the salivary flow rate and total salivary protein were not reported in this study. In addition, a significant correlation of salivary CRP with serum CRP (*r* = 0.599, *p* = 0.014) was demonstrated for individuals with high levels of serum CRP. Based on this finding, the study suggested that saliva can be used to measure inflammation, and CRP is a promising representative for systemic inflammation.

The same phenomenon is seen in a former study on the validation of salivary CRP in healthy adults that showed a moderate-to-strong correlation (*r* = 0.72, *p* < 0.001) of CRP found in saliva and serum, especially for subjects who have high serum CRP levels compared to those who have low serum CRP levels [67]. The study utilized the cut-off point of 3 mg/L for serum CRP levels (considered as high-risk for cardiovascular disease according to AHA/CDC guidelines [26]) and statistical equation to compute an estimation cut-off point for salivary CRP = 1629.39 pg/mL. The study also demonstrated association between high salivary CRP and high serum IL-6 levels (*r* = 0.30, *p* = 0.04) in subjects who smoke and who have a high body mass index (BMI).

The latest study has evaluated the detectability of salivary CRP in 35 infants (ranging from 23 to 42 weeks of age) compared to serum CRP [68]. The median salivary CRP concentration reported in the study was 3.1 ng/mL, which is much lower than adult salivary CRP concentrations that have been shown to be in the range of 35–217 pg/mL [59]. This may be due to the immature development of the immune system in young infants. Furthermore, the study also demonstrated a positive correlation between salivary and serum CRP in infants (*r* = 0.62, *p* < 0.001). The statistical analysis results showed that the optimal cut-off point for raw salivary CRP concentration at 4.84 ng/mL can be used to predict serum CRP of ≥10 mg/L and ≥5 mg/L, which had a corresponding sensitivity and specificity of “0.54 and 0.95” and “0.64 and 0.94,” respectively. This study suggested salivary CRP is potentially safe to be used as a routine screening assay for the at-risk newborn [68]. In our view, this study approach has potential to be extended to older population in order to determine the CRP threshold and to help to differentiate between normal and abnormal CRP level.

A number of validated commercial kits are available to capture the inflammatory response in saliva (Table 2). A few promising studies showed that CRP is a promising candidate as it has shown correlation of salivary levels with plasma. Further studies are necessary to correlate other salivary markers to blood. A study of inflammatory response to vaccination in saliva and blood would provide a perfect opportunity to understand the correlation of these markers in the two bodily fluids.

## 9. Conclusions and Outlook

Vaccination as an immune challenge could be relevant to identify individuals' inflammatory responses and immune status (immune fitness). Dynamic responses to a challenge provide a sensitive and meaningful indication of health. However, measuring inflammatory responses in blood might be difficult to cover the full dynamic range of inflammation. The aim of this review is to draw the attention to saliva as an alternative source to blood and serum. The saliva-based noninvasive approach brings several advantages over blood testing. The saliva collections are generally convenient and safe; moreover, multisample saliva collection provides more information than one single blood collection typically accessible, which could offer an opportunity to study the magnitude, time course, and the changes in inflammatory responses after vaccination.

To date, no published study has charted the time course of inflammatory responses to vaccination using saliva. Also, the kinetics and the mechanisms through which saliva components contribute to the inflammatory response after vaccination are not well understood. Hence, studies regarding the inflammation response to vaccination in saliva are needed to validate our proposed approach and to establish its value. Moreover, a standardization of experimental protocols should be established for vaccination inflammatory challenge studies.

## Figures and Tables

**Table 1:** Summary of studies on inflammatory response to vaccination.

Vaccination	Year	Studies	Number of subjects	Markers	Type of sample	Time course	Level observed	Results
DTwP/DTaP, Hib, HBV, IPV	1998	[19]	89 preterm infants	IL-6, CRP	Serum	0, 12, 36, 48 hours after injection	IL-6 (130 pg/mL) CRP (4.0 mg/dL)	IL-6: responded at the peak level at 12 hours after vaccination CRP: peak level at 36 hours after vaccination of DTwP but not DTaP, respectively

DT-Polio-Typhim	2005	[69]	7 elderly; 8 young adults	CRP, fibrinogen, albumin, *α*1-acid glycoprotein (AGP), al-antitrypsin, transthyretin and transferrin	Plasma	7 days before vaccination and 2 days after injection	CRP in elderly (7.1 mg/L); in young adults (11.5 mg/L)	CRP and AGP levels increased after 2 days after vaccination, (*p* < 0.05); no significant change in other acute-phase proteins

Hepatitis B	2013	[18]	70 infants	IL-6, CRP	Plasma	0 and 24 hours after injection	CRP (3.8 mg/L) IL-6 (11.4 pg/mL)	IL-6: no change (*p* > 0.05) CRP: level increased after 24 h (*p* < 0.001)

*Salmonella typhi*	2000	[70]	18 adults	Body temperature, IL-1*β*, IL-1Ra, IL-6, TNF*α*	Serum	0, 1, 2, 3, 4, 5, 6, 7, 8, and 32 hours after injection	IL-6 (5.8 pg/mL) IL-1Ra (593.6 pg/mL)	No change in body temperature; no change in IL-1*β* and TNF*α*; IL-1Ra level increased peak at 3 hours afterwards vaccination (*p* < 0.05); IL-6 level increased within 8 hours after vaccination (*p* = 0.07)
	2002	[71]	17 adults	CRP, IL-6, IL-1Ra, TNF-*α*	Plasma	0, 1, 2, 3, 4, 5, 6, 7, and 8 hours after injection	Not indicated	CRP: No significant changes within 8 hours after vaccination TNF-*α*: Level increased at 4 hours after vaccination IL-6: Level increased gradually for the first 4 hours and peaking at 4 hours after vaccination
	2005	[72]	30 male adults	IL-1Ra, IL6, TNF-*α*	Plasma	0, 3, and 6 hours after injection	IL-6 (1.59 pg/mL) IL-1Ra (682.92 pg/mL) TNF-*α* (248 pg/mL)	IL-6: level increased after 3 h IL-1Ra & TNF-*α*: No significant changes
	2013	[20]	8 male adults	CRP, IL-6, TNF-*α*	Plasma	0, 4, 5, 6, 7, 8, and 24 hours after injection	CRP (<0.1 mg/L) IL-6 (<6 pg/mL) TNF-*α* (<8 pg/mL)	IL6: peak at 6-7 h (*p* < 0.05) TNF-*α*: peak at 6–8 h (*p* = 0.034) CRP: level not increase within first 8 hours, perhaps peak after 24 h (*p* = 0.027)

Influenza	2005	[73]	22 adults	CRP, IL-6, MCP-1, TNF-*α*, IL-2sR*α*, and serum amyloid A (SAA)	Plasma	0, 1, 3, and 7 days after injection	Influenza CRP (1.4 mg/L) IL-6 (1.9 pg/mL)	CRP: level increased at day-1 (*p* < 0.01) and day-3 (*p* = 0.05) after vaccination IL-6: level increased peaking at day-1 after vaccination SAA: level increased at day-1 (*p* < 0.05) TNF-*α* and IL-2sR*α*: Not significant changes
	2011	[23]	46 pregnant women	CRP, IL-6, TNF-*α*	Serum	0, 1, 2, and 7 days after injection	CRP (0.85 mg/L) IL-6 (0.2 pg/mL) TNF-*α* (0.65 pg/mL)	CRP: level increased one and two days after vaccination (*p* < 0.05) TNF-*α*: level increased 2 days after vaccination (*p* = 0.06) IL-6: No significant change
	2013	[74]	28 pregnant & 28 nonpregnant women	IL-6, TNF-*α*, IL-1*β*, IL-8, MIF	Serum	0, 1, 2, and 3 days after injection	IL-6 (approx. 1.5 pg/mL) TNF-*α* (approx. 15.8 pg/mL) Il-1*β* (approx. 0.7 pg/mL) IL-8 (approx. 6–8 pg/mL) MIF (approx. 10 pg/mL)	TNF-*α* and IL-6: Level increased, peaking at one day after vaccination (*p* < 0.001); IL-1*β* and MIF: no significant change; IL-8: Level decreased in nonpregnant women; no change in pregnant women
Influenza; influenza + pneumococcal	2004	[22]	39 adults	CRP	Plasma	0, 2, or 3 days or 4 or 5 days after injection	Influenza CRP (2.7 mg/L) Influenza + pneumococcal CRP (3.7 mg/L)	CRP: level increased and peak at day 2 after vaccination

17D yellow fever	2002	[21]	10 adults	IL-6, CRP, fibrinogen	Plasma	0, 1, 2, 3, 4, 5, 6, 7, and 8 days after injection	IL-6 (0.33 pg/mL) CRP (1.20 ng/mL) Fibrinogen (2.91 g/L)	IL-6: level increased 30% on days 5 & 6 after injection (*p* < 0.05) CRP: level increased 45% on days 6 & 7 (*p* < 0.05) Fibrinogen: level increased 10% on day 2 and further arise on day 5 onwards (*p* < 0.05)

**Table 2:** Commercially available immunoassay (ELISA) for the detection of salivary markers.

Salivary markers	Companies	Serum-saliva correlation	Sensitivity
17*α*-Hydroxyprogesterone	Salimetrics	0.64	3 pg/mL
	DRG®	—	3 pg/mL
	Rocky Mountain Diagnostics	—	3.6 pg/mL
	Eagle Biosciences	—	4.9 pg/mL

Alpha-amylase	Salimetrics	—	0.4 U/mL
	MyBiosource.com	—	1.95 IU/mL
	Antibodies-onlines	—	0.94 ng/mL

Androstenedione	Salimetrics	0.77	5 pg/mL
	DRG®	—	5 pg/mL
	Eagle Biosciences	—	0.5 pg/mL

C-reactive protein	Salimetrics	—	10 pg/mL
	AbCam	—	0.25 ng/mL

Chromogranin	Salimetrics	—	0.7 ng/mL

Cortisol	Salimetrics	0.91	<0.007 *μ*g/dL
	DRG®	—	0.0537 *μ*g/dL
	Biomatik	—	<1.20 ng/mL
	Eagle Bioscience	—	0.025 ng/mL
	Rocky Mountain Diagnostics	—	0.014 ng/mL
	BioVendor Laboratory Medicine, Inc.	—	1.0 pg/mL

Cotinine	Salimetrics	—	0.15 ng/mL

DHEA	Salimetrics	0.86	5 pg/mL
	DRG®	—	3.3 pg/mL
	Rocky Mountain Diagnostics	—	2.2 pg/mL

DHEA-S	Salimetrics	—	43 pg/mL
	DRG®	—	25 pg/mL
	ALPCO	—	0.045 ng/mL
	Eagle Biosciences	—	0.05 ng/mL
	Rocky Mountain Diagnostics	—	0.045 ng/mL

Estradiol	Salimetrics	0.80	0.1 pg/mL
	DRG®	—	0.085 pg/mL
	Eagle Biosciences	—	0.5 pg/mL
	Rocky Mountain Diagnostics	—	0.4 pg/mL

Estriol	Salimetrics	0.87	1 pg/mL
	DRG®	—	2 pg/mL
	Biomatik	—	<0.03 ng/mL
	ALPCO	—	1.1 pg/mL
	Eagle Biosciences	—	0.5 pg/mL
	Rocky Mountain Diagnostics	—	1.1 pg/mL

Estrone	Salimetrics	—	1 pg/mL
	DRG®	—	4.74 pg/mL
	Biomatik	—	<1 pg/mL

Interleukin-6	Salimetrics	—	0.07 pg/mL

Interleukin-1 Beta	Salimetrics	—	<0.37 pg/mL

Melatonin	Salimetrics	0.81	1.37 pg/mL
	DRG®	—	0.3 pg/mL

Progesterone	Salimetrics	0.87	5 pg/mL
	Eagle Biosciences	—	4.9 pg/mL
	Biomatik	—	<20 pg/mL
	Rocky Mountain Diagnostics	—	3.8 pg/mL

Secretory Immunoglobulin A	Salimetrics	—	2.5 *μ*g/mL
	DRG®	—	0.5 *μ*g/mL

Testosterone	Salimetrics	0.96	1 pg/mL
	DRG®	—	1.9 pg/mL
	MyBiosource.com	—	1.9 pg/mL
	IBL	—	1.0 pg/mL
	Eagle Biosciences	—	2.96 pg/mL
	Biomatik	—	<1 pg/mL
	Rocky Mountain Diagnostics	—	1.9 pg/mL

TNF-*α*	Salimetrics	—	0.106 pg/mL
